# Supplementary material for: Seasonal variation in fish school spatial distribution and abundance under the Kuroshio regular pattern and the large meander in Suzu coastal waters
Source: PLoS One. 2021 Nov 30;16(11):e0260629. doi: 10.1371/journal.pone.0260629 (PMC8631671; doi:10.1371/journal.pone.0260629)
Supplement: Attachment — Submitted filename: Response to Reviewers.docx [file pone.0260629.s006.docx]

**Introduction**

**1. The Introduction needs to be restructured to improve its logicality.**

We have restructured the introduction. First, we introduced the main characteristics of the Kuroshio and why the Kuroshio is important for the local ecosystem. Next, we explained the two patterns of the Kuroshio and why we need to focus on the Kuroshio large meander (LM). Third, we introduced Tosa Bay, which is greatly affected by the Kuroshio Current, and set the net fishing industry in detail. Next, we explained why I had to use the acoustic method. Finally, the purpose of this study was pointed out.

**2. It was divided into three patterns of Kuroshio Current in Kaneko et al., 2019.**

The Kuroshio path south of Japan exhibits a remarkable bimodal feature: the large meander (LM) path and the non-large meander (NLM) path. The NLM path is sometimes further classified into two paths, namely, the offshore NLM (oNLM) and the nearshore NLM (nNLM).

[Usui, 2013 <https://link.springer.com/article/10.1007/s10872-013-0197-1>]

[Toida, 2019 <https://agriknowledge.affrc.go.jp/RN/2030927543.pdf>]

And, the offshore NLM (oNLM) and the nearshore NLM (nNLM) have the same effect on our study area. [Sugimoto, 2020 <https://doi.org/10.1007/s10872-019-00531-8>]. In the paper [Kaneko, 2019], the survey area is different from our study area. Therefore, there is only one type of NLM in our study area.

Study area in

(Kaneko, 2019)

Our study area

**3. A conceptual map of the Kuroshio is needed for readers who are not familiar with this area.**

The Kuroshio paths during the survey period (Fig 1) were drawn using data from Hydrographic and Oceanographic Department Japan Coast Guard. And water masses were modified from previous research. These information was added in the introduction.

**4. In my opinion, I think the Warm Current of Mexico Gulf is the largest warm current.**

We changed the sentence to “The Kuroshio Current is one of the warmest water currents in the world”.

**5. To my knowledge, the fishery of set-net is also largely dependent on the current that drive fishes into the cod-end. Although this paper focuses on the seasonal species composition, this should be declared in the introduction.**

We added the information about seasonal species composition with the change of Kuroshio Current in the introduction. As the sentence “In addition, the fish composition structure in Tosa Bay is seasonal, and the seasonal changes tend to differ depending on the berthing and undocking of the Kuroshio Current that year”.

**6. Can the large meander pattern last for several months? This should be clarified.**

From the previous researches, the large meander pattern appears once in a roughly ten-year cycle, and continues for more than 1 year. Additionally, the latest large meander occurred in August 2017 is the second longest in history.

[Nishiyama, 1980. <https://doi.org/10.2467/mripapers.31.43>]

[Miyama, 2019 <https://agriknowledge.affrc.go.jp/RN/2010927526.pdf>]

**Methods**

**7. The authors need to provide more details on model structures.**

The information about distribution family, linking function, smoothing methods, degree of freedom, explanatory and response variables was added in the methods.

**8. The reason for such analyses should be clarified as D and Db can used as explanatory variables in one group of GAM.**

We put the explanatory variables D and Db into one group, and performed a new analysis of the relationship with Sv.

**9. Model check process should be displayed to ensure the confidence on results, and the ‘gam.check’ routine is useful.**

We used the ‘gam.check’ to ensure the confidence on results. However, there are four result diagrams for one explanatory variable, and it is quite difficult to include the results of each season for two years for the three explanatory variables in the paper.

As an example, I posted one results for each response variable here.

201805_*Sv*

201705_*Sv*

201708_*Sa*

201608_*Sa*

Instead of these diagrams, the results about *p*-value, adjusted *r^2^*, Deviance explained (%) and GCV score were summarized in Table 5.

**10. Details on the measure of environmental factors are not well described.**

The marine environment (temperature and salinity) around the set net was measured for one day during each survey period using a RINKO-Profiler. Results of previous research are cited as supplementary information. Our survey results were not different from the monthly average water temperature for the same month.

[Ihara, 2019 <https://agriknowledge.affrc.go.jp/RN/2030927527.pdf>]

In addition, the temperature data from Kochi Prefecture Fishing Sea Condition Information System also shows that the difference in environmental factors was caused by different Kuroshio patterns instead of the different dates.

**Results and discussions**

**11. I strongly suggest that the author reorganize these results with focus on differences in species abundance, composition, and distribution.**

We have divided the results in three parts: 1) seasonal variations in fish abundance, 2) seasonal variations in fish composition, 3) seasonal variations in fish distribution, 4) seasonal variations in the marine environment, and focused on these difference between two patterns of Kuroshio Current.

For discussions, first, we talked about the marine environment affected by Kuroshio LM. Next, we discussed the fish species composition and abundance that changes due to the influence of the Kuroshio LM. After that, we discussed changes in fish school distribution due to changes in fish composition and abundance.

**12. “high fish school responses”. I do not understand this phrase.**

The word for the reflection intensity of fish school has been changed from "response" to "scatterer abundance". [Tournier, 2021 <https://doi.org/10.1016/j.jmarsys.2021.103608>]

**13. The English should be polished by native speakers to increase the readability**

This paper has been edited by editors from ‘Editage’ to ensure language and grammar accuracy. We have submitted an English proofreading certificate to PLOS ONE.
